# Synergistic Actions of Natural Compounds for Enhancing Cognitive and Physical Performance: A Narrative Review

**DOI:** 10.7759/cureus.102674

**Published:** 2026-01-30

**Authors:** Raghav Panda, Harvey N Mayrovitz

**Affiliations:** 1 Medical School, Nova Southeastern University Dr. Kiran C. Patel College of Osteopathic Medicine, Davie, USA; 2 Medical Education, Nova Southeastern University Dr. Kiran C. Patel College of Allopathic Medicine, Davie, USA

**Keywords:** beetroot, cordyceps, guayusa., l-citrulline, monk fruit, natural stimulants, niacin, nitric oxide, non-nutritive sweeteners, stevia

## Abstract

Energy drinks are widely consumed for cognitive and physical enhancement, yet most commercial formulations rely on a narrow mechanism: rapid stimulation from synthetic caffeine combined with artificial sweeteners. This stimulant-centric strategy overlooks the multidimensional physiology underlying sustained human energy, which emerges from interactions among neurocognitive activation, nitric oxide-mediated vascular function, mitochondrial ATP production, and metabolic homeostasis. This narrative review synthesizes evidence on naturally derived compounds that influence these pathways, including guayusa, guarana, *Alpinia galanga*, theobromine, L-citrulline, beetroot extract, cordyceps, and niacin, as well as natural sweeteners such as monk fruit and stevia. Evidence suggests that these agents may support cognitive performance, oxygen efficiency, vascular perfusion, and cellular energy capacity through mechanisms distinct from those of traditional energy drinks. However, heterogeneity across extract standardization, dosing, and study design limits the ability to draw definitive conclusions. A system-level understanding of energy physiology may guide the development of formulations better aligned with long-term cardiovascular, cognitive, and metabolic health.

## Introduction and background

Energy drink consumption has grown exponentially across adolescents, young adults, athletes, and shift workers, driven by demands for prolonged alertness and improved performance [1]. Despite this widespread use, most commercial formulations rely almost exclusively on synthetic caffeine as their functional backbone [2]. While caffeine is a well-established adenosine receptor antagonist, its effects are transient, dose-dependent, and often accompanied by adverse responses such as jitteriness, tachycardia, and post-stimulant fatigue [2,3]. Furthermore, artificial sweeteners, widely used to reduce caloric load, can influence metabolic signaling in ways that complicate energy homeostasis [4-8]. 

Human energy, however, is not governed by a single neurotransmitter or metabolic pathway. Instead, it emerges from interactions among neurocognitive activation, vascular nitric oxide (NO) signaling, mitochondrial ATP production, and metabolic regulation. Neurocognitive activation involves dopaminergic, cholinergic, and adenosinergic modulation, each shaping attention, vigilance, and mental clarity [3,9-12]. Vascular NO pathways optimize oxygen and nutrient delivery, a determinant of endurance and cognitive resilience under demanding conditions [13-19]. Mitochondrial ATP synthesis provides cellular energy currency, while metabolic homeostasis regulates glucose availability and insulin response, influencing both physical stamina and subjective fatigue [20-22]. 

Concerns about artificial sweeteners further highlight the need for alternative approaches. Sucralose, one of the most ubiquitous non-nutritive sweeteners, has been shown to alter glycemic responses, reduce insulin sensitivity, and induce hepatic insulin resistance [4-6]. Reviews note uncertainties regarding chronic endocrine effects [7], and preclinical studies suggest altered pancreatic insulin secretion [8]. These findings challenge common marketing claims and industry assumptions that certain artificial sweeteners are metabolically neutral. 

Given these complexities, naturally derived compounds with roles in neurological signaling, vascular regulation, mitochondrial function, and glycemic stability may offer broader physiological support. This review evaluates such compounds within a systems-level energy framework, emphasizing their mechanisms, evidence base, and potential relevance to performance and metabolic outcomes. 

Methods 

This narrative review aimed to synthesize current evidence on natural compounds that influence neurocognitive activation, nitric oxide-mediated vascular function, mitochondrial energy metabolism, and sweetener-related metabolic neutrality in the context of energy support. Electronic searches were conducted in PubMed, Embase, and Web of Science from database inception to October 2025. Full-text articles were obtained through institutional subscriptions and publicly available repositories, including PubMed Central (PMC), when accessible.

Search Strategy

The search strategy used combination of terms related to each compound and outcome: “guayusa” OR “Ilex guayusa," “Guarana,” “Alpinia galanga,” “theobromine,” “L-citrulline,” “citrulline malate,” “dietary nitrate,” “beetroot juice,” “Cordyceps militaris,” “Cordyceps sinensis,” “niacin” AND “NAD,” “monk fruit,” “stevia,” and “sucralose metabolism.” Boolean operators and additional keywords such as “cognition,” “attention,” “reaction time,” “VO₂ kinetics,” “endurance,” “exercise performance,” “mitochondrial function,” “insulin sensitivity,” and “glycemic control” were used to refine search results. Reference lists of included articles and relevant reviews were hand-searchedto identify additional publications. 

Eligibility Criteria

Inclusion criteria were: (i) peer-reviewed human or animal studies; (ii) mechanistic, preclinical, or clinical investigations evaluating one or more of the target compounds; and (iii) reported outcomes related to neurocognitive function, vascular or nitric oxide pathways, mitochondrial or cellular energy metabolism, or glycemic and metabolic effects. Narrative reviews, systematic reviews, and meta-analyses were consulted for background and context but were not treated as primary evidence. 

Exclusion criteria included non-peer-reviewed sources, conference abstracts without full articles, case reports, studies focused solely on unrelated therapeutic indications (for example, oncology outcomes without energy- or metabolism-related endpoints), and articles not available in English. Studies of proprietary blends in which the identity of the dose of key ingredients could not be determined were also excluded. 

Study Selection and Data Synthesis

Titles and abstracts identified by the search were screened for relevance by the primary author. Full texts of potentially eligible studies were reviewed in detail, and uncertainties about the inclusion were resolved through discussion with the senior author. Because of heterogeneity in study designs, populations, dosing regimens, and outcome measures, no formal risk-of-bias tool or quantitative meta-analysis was applied. Instead, data were synthesized qualitatively and organized into four mechanistic domains: (i) neurocognitive activation, (ii) nitric oxide-mediated vascular responses, (iii) mitochondrial energy metabolism, and (iv) sweetener-related metabolic neutrality. 

## Review

The neurocognitive activation pathway 

*Guayusa (Ilex guayusa)* 

Guayusa is a naturally caffeinated Amazonian leaf traditionally consumed as an herbal infusion [23]. It contains caffeine, theobromine, chlorogenic acids, and polyphenols, resulting in a distinct phytochemical profile that modulates stimulant kinetics [23,24]. The United States Food and Drug Administration (FDA) recognizes guayusa extract as Generally Recognized as Safe (GRAS) [23], and toxicological evaluations support its safety [25]. Compared with synthetic caffeine, guayusa has been associated with smoother stimulant effects, possibly due to theobromine’s longer half-life and its vasodilatory properties [3,24,25]. Chlorogenic acids may further influence glucose regulation and neuronal excitability. Recent processing studies show that guayusa’s antioxidant load, meaning the total concentration of antioxidant compounds capable of reducing oxidative stress, is substantial and varies depending on the extraction technique [26]. Antioxidants may contribute to reduced oxidative stress during cognitive load, potentially supporting mental clarity under sustained performance conditions [26]. 

*Guarana (Paullinia cupana)* 

Guarana is an Amazonian seed traditionally used for alertness and energy due to its naturally high caffeine content [23,24]. Guarana seeds provide caffeine complexed with tannins, slowing absorption and prolonging stimulant effects [3,24]. Clinical studies demonstrate improved secondary memory, attentional accuracy, and psychomotor performance relative to caffeine alone. Polyphenol-rich extracts of guarana have been shown in controlled studies to enhance cerebral blood flow and exert anti-inflammatory effects, which may contribute to smoother cognitive stimulation [3,23-26]. As with other natural caffeine sources, excessive intake may lead to adverse effects, including jitteriness, tachycardia, and sleep disruption, particularly at total daily caffeine intakes exceeding approximately 400 mg in healthy adults [2]. Such effects may be more pronounced when caffeine is combined with other stimulants.

Substances impacting the neurocognitive activation pathway 

Greater Galangal (Alpinia galanga)

*Alpinia galanga *is a botanical root in the ginger family traditionally used to enhance alertness and relieve fatigue [27-29]. Randomized controlled trials have shown that *Alpinia galanga* enhances mental alertness, attention, and cognitive processing speed [27]. Additional research notes improvements in reaction time, focus, and resistance to mental fatigue [28-30]. Clinical trials consistently report that these cognitive effects occur with minimal cardiovascular stimulation [27]. Mechanistic studies suggest these effects may be mediated through dopaminergic and cholinergic pathways [30], supported by both clinical and preclinical evidence [27-31]. Preclinical models demonstrate antioxidant and neuroprotective actions, suggesting potential relevance for cognitive resilience during prolonged mental tasks [30].

Theobromine 

Theobromine is a natural compound found in cacao that provides a mild stimulant effect without the intensity of caffeine. It produces mild CNS stimulation while reducing peripheral vasoconstriction. Behavioral studies show improvements in mood, alertness, and cognitive steadiness when doses of 100-250 mg are administered in controlled laboratory conditions [3]. Its slower pharmacokinetics compared with caffeine may reduce the likelihood of abrupt “crash” phenomena [32]. Controlled vascular studies demonstrate that theobromine increases cerebral blood flow via nitric oxide-mediated vasodilation, supporting its potential role in cortical activation [3,32]. At doses substantially exceeding those typically consumed from dietary sources or evaluated in controlled studies (generally ≤250 mg), theobromine has been associated with mild gastrointestinal discomfort, restlessness, and sleep disturbance, particularly when consumed in combination with other methylxanthines. Controlled human studies indicate good tolerability within commonly studied dose ranges [3].

Substances impacting the nitric oxide-mediated vascular pathway 

L-Citrulline 

The amino acid L-citrulline bypasses hepatic metabolism and increases circulating L-arginine more effectively than dietary arginine [13]. Enhanced NO availability improves vasodilation, oxygen delivery, and muscle perfusion [13,14]. A meta-analysis reported increased aerobic performance, reduced fatigue, and improved endurance across multiple exercise modalities when 6-8 grams of L-citrulline malate were taken approximately one hour before exercise [14]. Bailey et al. demonstrated improved VO₂ kinetics, reflecting more efficient oxygen utilization during exercise transitions [15]. This effect was observed using a 6-gram dose of L-citrulline malate administered before exercise [15]. Strength-endurance trials also show increased repetitions and reduced perceived exertion, suggesting benefits for both aerobic and anaerobic performance. Studies evaluating strength-endurance performance typically used 8 grams of L-citrulline malate taken 60 minutes before exercise [16]. 

Beetroot Extract 

Beetroot is one of the most potent dietary nitrate sources . Through the nitrate → nitrite → NO pathway, beetroot can increase NO bioavailability under both normoxic and hypoxic conditions . Reviews indicate that dietary nitrate supplementation, typically providing ≥300 mg of nitrate equivalents (commonly via beetroot juice) and consumed approximately two to three hours prior to exercise, is associated with reductions in the oxygen cost of exercise and improvements in exercise tolerance and time-to-exhaustion [17]. Exercise trials show increased cardiorespiratory efficiency and improved high-intensity performance with nitrate doses in the 400-600 mg range, taken two to three hours pre-exercise during controlled endurance and interval training  [18]. Peripheral effects include improved microvascular perfusion, mitochondrial efficiency, and reduced ATP turnover during moderate- to high-intensity exercise following ingestion of 300-600 mg nitrate equivalents several hours before activity [19]. Dietary nitrate supplementation at doses commonly used in exercise studies (approximately 300-600 mg nitrate equivalents) is generally well tolerated [28]. Transient gastrointestinal discomfort or reductions in blood pressure may occur in susceptible individuals, particularly when combined with antihypertensive medications or phosphodiesterase-5 inhibitors [28].

Substances impacting the mitochondrial energy metabolism pathway 

*Cordyceps* 

Cordyceps is a type of medicinal mushroom traditionally used in Eastern medicine to support energy, stamina, and respiratory function [6,7,9]. It contains bioactive molecules such as cordycepin and adenosine analogs that may influence mitochondrial energy production [8]. Clinical trials have documented improvements in aerobic performance, VO₂ max, and fatigue resistance at daily doses of 1-3 grams of Cordyceps militaris extract, administered orally for two to six weeks [6-9]. Mechanistic studies suggest that cordyceps enhances activities of cytochrome c oxidase and other mitochondrial enzymes, potentially improving oxidative phosphorylation efficiency. Additional evidence indicates that cordyceps may modulate AMP-activated protein kinase (AMPK) signaling, a key regulator of cellular energy balance [8]. 

Niacin (Vitamin B3) 

Niacin is central to NAD⁺ biosynthesis, which supports mitochondrial ATP production and drives redox reactions. NAD⁺ influences sirtuin activation, mitochondrial biogenesis, and genomic stability [24]. Reviews show that niacin supplementation can enhance metabolic flexibility and mitochondrial resilience under physiological stress at oral doses of 250-500 mg/day of nicotinamide riboside or nicotinamide mononucleotide for 2-12 weeks in controlled human studies [24]. A WHO-linked review highlights its importance for neurological and metabolic function [26]. Recent human studies show that dietary niacin increases NAD⁺ metabolites and improves markers of cellular energy capacity [27,28]. These pathways are especially relevant in conditions of high metabolic demand. At pharmacologic doses, niacin is known to cause a transient cutaneous flushing response, which commonly occurs at intakes above approximately 30-50 mg/day of nicotinic acid [21]. Higher doses (>500 mg/day) have been associated with hepatic or glycemic effects; however, these outcomes are dose-dependent and uncommon at intake levels typically used in nutritional and metabolic studies [21,23].

Sweeteners and metabolic neutrality 

Concerns with Sucralose 

Sucralose is commonly used in energy beverages due to its high sweetness intensity and zero caloric contribution. However, repeated human studies show that it can significantly alter glycemic responses, reduce insulin sensitivity, and impair glucose tolerance [1-3]. Reviews highlight uncertainties regarding effects on gut hormones and microbiota [4]. Preclinical models indicate altered pancreatic insulin secretion and disruptions in hepatic insulin signaling [5]. These findings raise concerns about chronic use, particularly in individuals consuming multiple servings of energy drinks daily. 

*Natural Sweeteners* 

Extracts from the subtropical melon, monk fruit, provide sweetness via mogrosides, which have been shown to improve glycemic responses without stimulating insulin secretion [29]. Stevia glycosides found in Stevia plants exhibitantioxidant activity and minimal impact on glycemic control [30]. Their metabolic neutrality makes them attractive alternatives in formulations seeking to avoid insulin dynamics that may counteract energy-supportive pathways. 

Discussion 

This review examined natural compounds across multiple physiological domains relevant to energy. Neurocognitive enhancers such as guayusa, guarana, A. galanga, and theobromine support attention, alertness, and mental endurance through balanced methylxanthine-polyphenol interactions [17-23,31-33]. NO-enhancing compounds (L-citrulline and beetroot extract) consistently improve oxygen kinetics and endurance, with well-documented benefits across exercise modalities [10-16]. Mitochondrial-supportive agents (cordyceps and niacin) influence ATP production and metabolic resilience, supporting energy demands during sustained physical or cognitive activity [6-9,24,26-28]. 

Importantly, the choice of sweetener significantly influences metabolic outcomes. Sucralose’s effects on insulin sensitivity and glucose tolerance contrast with monk fruit and stevia’s metabolic neutrality [1-5,29,30]. As energy drinks are often consumed multiple times per day, cumulative metabolic effects warrant consideration. 

Although individual mechanisms are promising, few studies assess multi-ingredient combinations. The limited studies evaluating multi-ingredient formulations report modest improvements in perceived energy, cognitive alertness, and exercise tolerance, although effects vary based on ingredient composition and dosing. Theoretically, combining ingredients that target distinct physiological pathways may produce additive or synergistic effects; the following points represent speculative examples rather than established outcomes. Neurocognitive stimulants could enhance vigilance and reaction time. NO-boosting agents could improve oxygen delivery to working tissues. Mitochondrial enhancers could increase cellular ATP yield.

Metabolically neutral sweeteners may prevent glycemic disturbances that undermine energy stability. Such multi-pathway interactions could produce additive or synergistic effects, but this remains untested and should be a priority for future research. 

**Figure 1:**
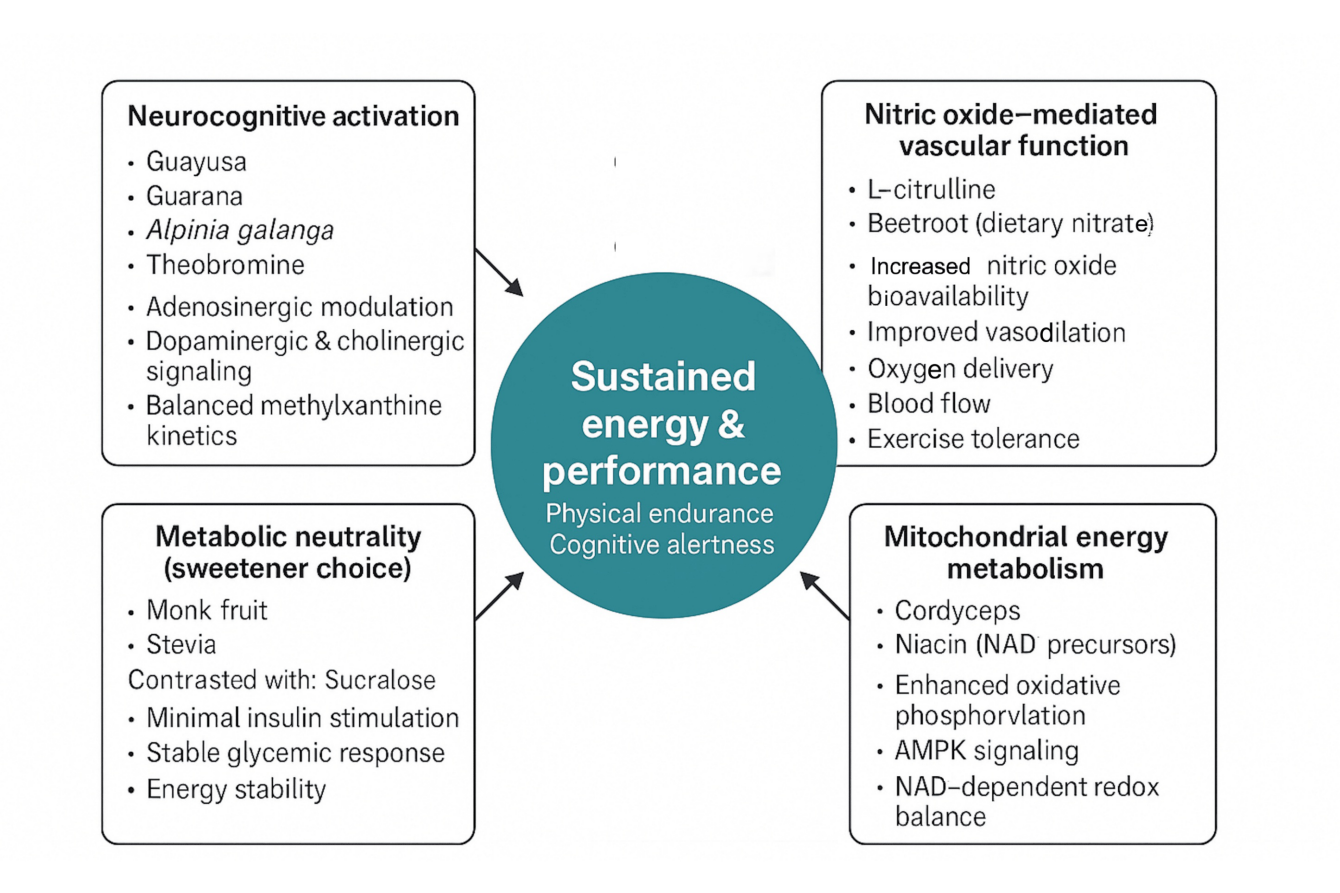
Systems-level pathways contributing to sustained energy and performance. The figure illustrates four complementary physiological domains that support energy and performance: neurocognitive activation, nitric oxide–mediated vascular function, mitochondrial energy metabolism, and metabolic neutrality. Neurocognitive activation involves modulation of adenosinergic, dopaminergic, and cholinergic signaling to support alertness and focus. Nitric oxide–mediated vascular function enhances blood flow, oxygen delivery, and exercise tolerance. Mitochondrial energy metabolism supports ATP production and cellular energy efficiency through oxidative phosphorylation and NAD⁺-dependent pathways. Metabolic neutrality reflects sweetener choices that minimize insulin stimulation and glycemic disruption, supporting energy stability. Together, these pathways converge to promote sustained physical and cognitive performance. Image Source: Authors

Figure 1 illustrates the components and interactions that contribute to sustained energy and performance, as described in this review.

Limitations and mixed findings in the literature

Despite substantial evidence supporting nitric oxide-mediated and stimulant-related mechanisms for enhancing performance, findings across studies are not uniformly positive. Controlled trials of dietary nitrate supplementation have reported no significant improvements in endurance performance under certain physiological or environmental conditions, including cycling time-trial performance in the heat, despite confirmed increases in nitrate and nitrite biomarkers [34]. These findings suggest that ergogenic efficacy may be context-dependent and influenced by factors such as thermoregulation, training status, and baseline nitric oxide bioavailability.

Similarly, investigations of stimulant-related cognitive enhancement indicate that subjective improvements in alertness or perceived attention do not always translate into measurable gains in objective cognitive performance. Comprehensive reviews of caffeine-based interventions report considerable variability across cognitive domains, with mood and vigilance often improving independently of reaction time, executive function, or sustained attention outcomes [35]. In parallel, heterogeneity in physiological responsiveness to dietary nitrate has been widely documented, with responder-nonresponder patterns and methodological differences contributing to mixed outcomes across trials [36]. Together, these mixed findings underscore the importance of cautious interpretation of single-pathway interventions and reinforce the need for rigorously controlled studies that integrate both objective physiological measures and perceptual outcomes.

## Conclusions

While findings across individual compounds are promising, reported effects are not uniform across studies, and observed benefits appear sensitive to dosing, study design, and participant characteristics. Human energy regulation reflects the coordinated function of neurocognitive, vascular, mitochondrial, and metabolic systems rather than the activation of a single stimulatory pathway. The natural compounds reviewed in this paper engage these physiological domains through mechanisms that differ from those of conventional energy formulations, which commonly emphasize acute stimulant effects. By influencing neurotransmitter signaling, nitric oxide-dependent vascular responses, mitochondrial energy metabolism, and metabolic stability, these compounds may offer an alternative framework for supporting energy-related outcomes. Concerns regarding the metabolic effects of artificial sweeteners further highlight the importance of evaluating alternative sweetening strategies that minimize glycemic disruption while maintaining consumer acceptability.

Although existing evidence for individual ingredients suggests potential benefits, the majority of studies are limited by short durations, controlled settings, or single-ingredient designs. Consequently, the clinical relevance of these findings to combined formulations and habitual use remains uncertain. Future trials should focus on rigorously designed, long-term clinical trials that evaluate multi-ingredient formulations, assess safety alongside efficacy, and examine outcomes across diverse populations and use contexts. A systems-level perspective that considers interactions among neurological, vascular, mitochondrial, and metabolic pathways may help inform the development and evaluation of energy-support strategies that align more closely with human physiology and clinical considerations.
